# College-to-NFL Stadium Turf Transitions as a Risk Factor for Lower Extremity Non-Contact Injuries in Rookie Players: A 13-Year Cohort Analysis

**DOI:** 10.3390/healthcare13121415

**Published:** 2025-06-13

**Authors:** Bahman Adlou, John Grace, Christopher Wilburn, Wendi Weimar

**Affiliations:** Sport Biomechanics Lab, School of Kinesiology, Auburn University, 301 Wire Rd, Auburn, AL 36830, USA; jlg0068@auburn.edu (J.G.);

**Keywords:** National Football League (NFL), lower extremity injury, non-contact injury, playing surface, artificial turf, rookie athletes, risk factors

## Abstract

**Background/Objectives**: Lower extremity non-contact injuries (LE-NCIs) pose a significant burden on the National Football League (NFL), with ongoing debates regarding playing surface safety. The stressful college-to-professional transition period for rookies, which can include adapting to new playing surfaces, may influence injury susceptibility. This study aimed to determine whether the transition in a home stadium turf type (natural grass, artificial, and hybrid) from the final college season to the rookie NFL season impacts LE-NCI likelihood. **Methods**: A retrospective cohort study analyzed 826 first and second-round NFL draft picks from 2012 to 2024. Data on college/NFL home surfaces (defining six transition types), position group, college training surface access, and rookie season LE-NCIs were collected from public sources. Competing risk analysis was used to estimate the cumulative LE-NCI incidence. Multivariable logistic regression assessed the association between turf transition and LE-NCI risk, adjusting for position, draft cohort, and college training access. **Results**: During their rookie season, 21.2% (175/826) of players sustained an LE-NCI. Skill position players had significantly higher adjusted odds of LE-NCI compared to hybrid players (AOR = 1.88; 95% CI: 1.20–2.97; *p* = 0.006). No specific turf transition category showed a statistically significant association with LE-NCI risk compared to the Grass-to-Grass reference in adjusted models. College training surface access was also not significantly associated with risk (AOR = 0.97; 95% CI: 0.65–1.45; *p* = 0.874). Cumulative LE-NCI incidence reached 33.1% by season end, with risk accelerating between weeks 4 and 10. **Conclusions**: Home stadium turf-type transition from college to the NFL was not significantly associated with LE-NCI risk in this rookie cohort, suggesting that surface transitions may not be a primary risk factor during the professional transition period. However, our analysis revealed significant position-dependent injury patterns (skill players: AOR = 1.88) and a temporal clustering of injuries between weeks 4 and 10, indicating that rookie LE-NCI prevention strategies should prioritize position-specific interventions and enhanced monitoring during the early- to mid-season high-risk period rather than surface transition-based approaches.

## 1. Introduction

Lower extremity non-contact injuries (LE-NCIs) remain a significant concern in professional field sports, profoundly impacting player careers, team performance, and healthcare costs [1]. The relationship between playing surface type and injury risk has emerged as a contentious issue in the National Football League (NFL), with players, medical staff, and administrators’ ongoing debate regarding the comparative safety of artificial turf versus natural grass [1,2].

Recent analyses have intensified this debate, with the NFL Players Association formally advocating for natural grass transitions based on injury surveillance data [3,4]. Studies examining the 2021–2022 NFL seasons found higher lower extremity injury rates on artificial turf (1.42 vs. 1.22 injuries per game) and significantly increased odds of season-ending surgery (OR = 1.60; 95% CI: 1.28–1.99) [1]. Data from 2020 to 2023 indicate that artificial surfaces, representing 56.8% of games, were associated with 61.1% of knee ligament tears [2].

Natural grass, artificial turf, and emerging hybrid surfaces exhibit distinct physical and mechanical properties that directly influence athlete–surface interactions. These surface differences influence lower extremity biomechanics through three critical mechanisms: traction, compliance/stiffness, and energy restitution. Natural grass surfaces demonstrate variable compliance (10–15 mm surface deformation) and rotational resistance (35–45 Nm rotational torque) that fluctuates with environmental conditions [5]. Modern artificial turf systems exhibit 15–22% higher rotational resistance and reduced compliance variability, with surface stiffness ranging from 60 to 85 Shore A hardness depending on infill composition [6]. Excessive rotational traction can “fix” the foot during directional changes, transferring high injury risk torque to ankle and knee joints while on grass [7]. Increased surface stiffness elevates ground reaction forces by 15–25%, altering joint kinetics and potentially increasing ligamentous strain [8]. Hybrid reinforcement systems display intermediate characteristics, combining natural variability with synthetic durability.

Athletes develop surface-specific neuromuscular control strategies through repeated exposure. Natural grass variability requires continuous proprioceptive adaptation, engaging dynamic stabilization mechanisms [9], while artificial turf uniformity may reduce stride-to-stride neuromuscular adjustments [10]. This neuromotor distinction highlights a critical hypothesis: abrupt surface transitions could disrupt established movement patterns, creating vulnerability windows during the college-to-professional transition period. A sudden change in the predominant playing surface—from college to professional settings—necessitates the recalibration of complex neuromechanical systems. This adaptation period could represent a window of heightened vulnerability to LE-NCIs, particularly while rookies simultaneously manage the substantial physical, psychological, and environmental stressors inherent to the college-to-professional transition [11,12].

This study examined whether home stadium turf transitions from college to the NFL impact LE-NCI likelihood in first and second-round draft picks (2012–2024). We hypothesized that (1) surface transitions would increase LE-NCI risk compared to stable Grass-to-Grass transitions; (2) varied college training surface access would be protective; and (3) the position group would moderate transition effects based on movement demands.

Understanding surface transition effects has significant implications for injury prevention protocols, player advocacy, and facility design policies, potentially reducing LE-NCI burden while improving player safety and performance quality.

## 2. Materials and Methods

### 2.1. Study Design and Population

This retrospective cohort study examined NFL players drafted in the first and second rounds between 2012 and 2024. We focused on these high-round draft picks as they typically receive substantial playing time during their rookie seasons, providing adequate exposure to evaluate injury risk. Additionally, these players represent significant financial and strategic investments for teams, making their injury prevention particularly important from organizational and league perspectives. The initial cohort included 827 players. After position group categorization by biomechanical position demands [13], one participant from the specialist position group (a kicker) was excluded due to being the sole representative in that category, resulting in a final analytical sample of 826 players.

### 2.2. Data Sources and Collection

Data were obtained from multiple sources, including Pro-Football-Reference [14] and NFL.com for injury reports, as well as official team, league websites, and publicly available reports from news archives from credible sources (NFL.com, ESPN.com, and CSB Sports) to complete information about injury conditions (e.g., surface, game vs. practice, etc.). This approach of using multiple data sources for cross-verification is consistent with previous NFL injury research methodologies. For each player, we collected demographic information (draft year, draft round, college, and rookie NFL team) and categorized their playing position into one of four position groups based on similar physical demands and movement patterns previously established for this population [13]:

- Linemen (305 players, 36.9%): Offensive and defensive linemen, defensive ends, defensive tackles, guards, tackles, centers, and nose tackles;
- Skill (291 players, 35.2%): Wide receivers, running backs, defensive backs, cornerbacks, and safeties;
- Hybrid (177 players, 21.4%): Linebackers (inside, outside), tight ends, and fullbacks;
- Quarterbacks (53 players, 6.4%).

Information regarding college and NFL home stadium playing surfaces was gathered from official stadium specifications and team websites and verified through published stadium reports. Surface types were classified as natural grass, artificial turf, or hybrid systems (specialized systems combining natural grass with synthetic reinforcement). This classification yielded six transition categories from college to NFL: Grass-to-Grass (G-G, 24.0%), Grass-to-Artificial (G-A, 24.3%), Artificial-to-Grass (A-G, 23.5%), Artificial-to-Artificial (A-A, 21.1%), Grass-to-Hybrid (G-H, 3.4%), and Artificial-to-Hybrid (A-H, 3.8%). Hybrid surface types were exclusive to professional NFL teams.

Additionally, we collected data on whether players had access to different turf types at their college training facilities during their final collegiate season, coded as a binary variable (yes, 70.6%; no, 29.4%). This variable was included to assess whether exposure to multiple surface types during training might affect injury adaptation.

“Draft Cohort” (2012–2015, 2016–2019, and 2020–2024) was included to account for temporal changes in playing surfaces, injury reporting, and player development protocols. The draft cohort groupings were based on an analysis of injury trends, surface technology adoption timelines, and NFL policy changes, with each period representing distinct eras in playing surface evolution: Early Infill Turf Era (2012–2015), Hybrid Surface Transition (2016–2019), and Turf 4.0/Grass Revival (2020–2024).

The primary outcome measure was the occurrence of the first lower extremity non-contact injury (LE-NCI) during a player’s rookie NFL season, encompassing both preseason and regular season periods. LE-NCIs were defined as injuries to the toe and foot, ankle, lower leg, knee, thigh, hip, and lower back occurring without direct contact from another player or object.

### 2.3. Statistical Analysis

Descriptive statistics were calculated for all variables. Categorical variables were presented as frequencies and percentages. Initial exploratory data analysis involved characterizing the entire cohort (*N* = 826) and examining the distribution of injury types, locations, and playing surfaces among injured players.

Bivariate associations between predictor variables and LE-NCI occurrence were assessed using Chi-square tests or Fisher’s exact tests (when expected cell counts were <5).

To understand how injuries develop over the rookie season, we tracked when players experienced their first lower extremity non-contact injury (LE-NCI). We needed to account for the fact that some players might experience other types of injuries first (like concussions or upper body injuries), which would temporarily or permanently remove them from the risk of sustaining an LE-NCI. To handle this complexity, we used a specialized statistical method (the non-parametric Aalen–Johansen estimator) that calculates the true probability of experiencing an LE-NCI by each week of the season.

In our analysis, we considered three possible scenarios for each player: (1) the player experienced an LE-NCI (our primary interest), (2) the player experienced a different injury first (competing event), or (3) the player completed the season without any injury (these players’ data were “censored,” meaning we only know they remained injury-free for the observed period). This approach allowed us to generate weekly estimates of injury risk throughout the rookie season while accounting for players who were removed from risk due to other injuries. When multiple injuries occurred during the same week, we applied a minor statistical adjustment (random jittering) to properly sequence these events.

Multivariable logistic regression models were constructed to estimate adjusted odds ratios (AORs) with 95% confidence intervals for the association between turf transition and LE-NCI risk. Two models were developed:

- Model 1: This included turf transition (with G-G as the reference category), position group (with hybrid as the reference category), and draft cohort (with 2012–2015 as the reference category) as predictors.
- Model 2: This was built upon Model 1 by adding college training access as a predictor.

An interaction term between turf transition and position group was initially considered to assess effect modification but was ultimately not included in the final models due to data sparsity in certain strata, which resulted in model instability. Cross-tabulation of transition types by position groups revealed several cells with very few cases, particularly when further stratified by injury outcome.

A sensitivity analysis was conducted using a subset of players comparing only those with LE-NCIs (*n* = 175) to those who remained uninjured throughout the season (*n* = 267), excluding players with other types of injuries. This approach allowed us to assess whether the observed associations were robust when using a more restrictive comparison group.

Players with missing outcome data were excluded from regression analyses (complete case analysis for the dependent variable). Missing values in categorical predictors were handled by including an “Unknown” category where appropriate for descriptive analysis; for regression models, missingness was minimal and did not materially affect the analytical sample.

All statistical analyses were performed using Python version 3.12. Statistical significance was defined as *p* < 0.05 (two-tailed).

## 3. Results

### 3.1. Sample Characteristics

The analytical sample consisted of 826 first and second-round NFL draft picks from 2012 to 2024 after excluding one specialist (kicker) due to insufficient representation in that position group. The sample included linemen (*n* = 305, 36.9%), skill positions (*n* = 291, 35.2%), hybrid positions (*n* = 177, 21.4%), and quarterbacks (*n* = 53, 6.4%). Overall, 559 players (67.6%) experienced some form of injury during their rookie season, with 175 players (21.2%) specifically sustaining a lower extremity non-contact injury. Among all injured players, the distribution of injury types was as follows: contact injuries (34.5%), non-contact injuries (33.5%), and unspecified mechanisms (32.0%).

**Figure 1:**
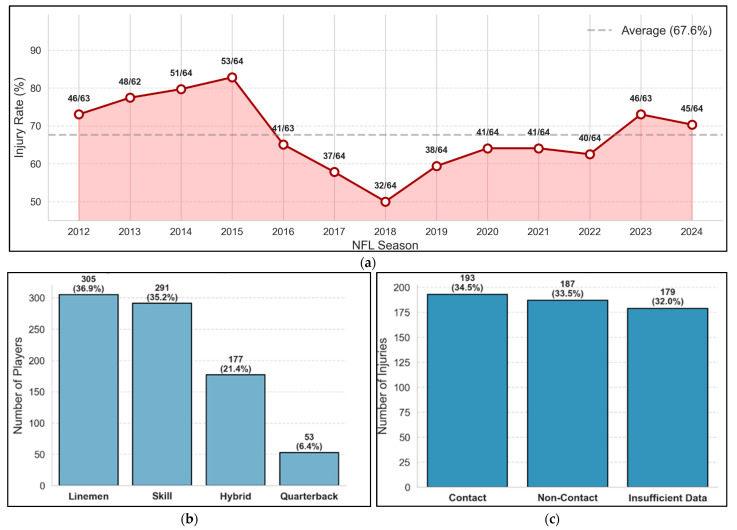

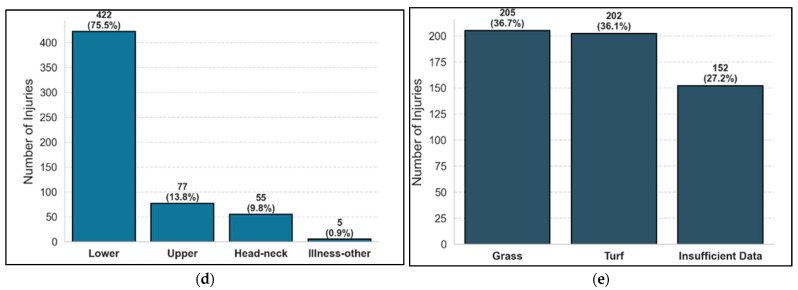
Descriptive statistics of NFL rookie cohort (*n* = 826). (**a**) Overall injury rate (%) by season (2012–2024), annotated with injured/total players per season. (**b**) Distribution of players by position group, *n* (%). (**c**) Distribution of injury mechanisms among injured players (*n* = 559), *n* (%). (**d**) Distribution of anatomical injury locations among injured players (*n* = 559), *n* (%). (**e**) Distribution of playing surfaces at time of injury among injured players (*n* = 559), *n* (%).

Among all injured players, injuries were categorized as contact injuries (34.5%), non-contact injuries (33.5%), and unspecified mechanisms (32.0%). Anatomically, injuries predominantly affected the lower extremity (75.5%), followed by the upper extremity (13.8%) and the head/neck region, including concussions (9.8%). Within LE-NCI athletes, injuries occurred during regular season games (32.0%), practice sessions (32.0%), preseason practices (5.1%), and preseason games (0.6%); additionally, there were two cases of college-season injury before draft, and the remaining 29.7% lacked setting documentation. The distribution of LE-NCIs by playing surface showed occurrences on natural grass (46.9%) and artificial turf (44.0%), with 9.1% lacking surface documentation (Figure 1).

### 3.2. Bivariate Associations

A Chi-square analysis revealed a significant association between position group and LE-NCI occurrence (χ^2^ = 20.1, *p* < 0.001). Skill position players had the highest injury rate (30.2%, 88/291), followed by hybrid players (19.2%, 34/177), linemen (15.1%, 46/305), and quarterbacks (13.2%, 7/53). Table 1 presents the baseline characteristics stratified by LE-NCI status.

**Figure 2:**
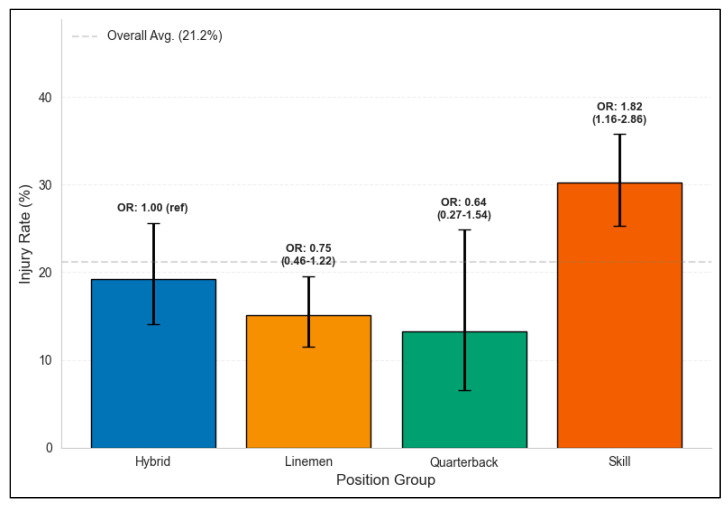
Lower extremity non-contact injury rates by position group. Lower extremity non-contact injury (LE-NCI) rates among NFL rookies (2012–2024, Rounds 1–2) by position group. Bars represent percentage of players within each group who sustained LE-NCI during their rookie season. Error bars indicate 95% Wilson score confidence interval for rate. Text above bars shows odds ratio (OR) for LE-NCI compared to hybrid group (reference) in Chi-square analysis. OR < 1 for specific position group indicates that players in that group have lower odds of experiencing lower extremity non-contact injury compared to players in hybrid group. Dashed line represents overall average LE-NCI rate (21.2%) across all included position groups. Difference in injury rates across groups was statistically significant (Chi-square test, *p* < 0.001).

The variation in LE-NCI rates across surface transition types was not statistically significant (*p* = 0.175): G-G (23.7%, 47/198), G-A (21.9%, 44/201), A-G (24.2%, 47/194), A-A (17.8%, 31/174), G-H (7.1%, 2/28), and A-H (12.9%, 4/31). College training access to multiple surface types was not significantly associated with LE-NCI occurrence (*p* = 0.923), with similar injury rates being observed among those with access (21.1%, 123/583) and those without (21.4%, 52/243; Figure 2).

### 3.3. Cumulative Incidence Analysis

Using the Aalen–Johansen estimator to account for competing risks, the cumulative incidence of LE-NCIs reached 33.1% (95% CI: 28.7–37.5%) by the end of the rookie season (week 18). The incidence curve showed an acceleration between weeks 4 and 10, followed by a more gradual increase until the season’s conclusion.

### 3.4. Multivariable Analysis

Table A1 presents the complete results of two multivariable logistic regression models examining the association between turf transition and LE-NCI risk. In Model 1 (including turf transition, position group, and draft cohort), no surface transitions showed statistically significant associations with LE-NCI occurrence compared to the Grass-to-Grass reference group, though the Grass-to-Hybrid transition showed the lowest adjusted odds ratio (AOR = 0.25, 95% CI: 0.06–1.12, *p* = 0.071). Position group was significantly associated with injury risk, with skill position players showing higher odds of LE-NCI compared to hybrid players (AOR = 1.88; 95% CI: 1.20–2.97; *p* = 0.006).

In Model 2, which additionally included college training access, the associations between turf transitions and position groups with LE-NCI remained consistent with Model 1. Access to multiple training surfaces during the final college season was not significantly associated with LE-NCI risk (AOR = 0.97; 95% CI: 0.58–1.48; *p* = 0.874).

### 3.5. Position-Specific Analysis

Table 2 presents detailed position-specific injury patterns across all surface transition categories. Relative to hybrid players (19.2% injury rate), skill position players had a significantly higher LE-NCI risk (30.2%; AOR = 1.88; 95% CI: 1.20–2.97; *p* = 0.006). Linemen (15.1%; AOR = 0.76; 95% CI: 0.47–1.25) and quarterbacks (13.2%; AOR = 0.64; 95% CI: 0.26–1.54) showed lower though not statistically significant risk differences compared to hybrid players.

Within position groups, notable patterns emerged across transition categories. Skill players transitioning from Grass-to-Grass surfaces had both the highest absolute number of LE-NCIs (*n* = 27) and one of the highest injury rates (38.6%), while those transitioning from Artificial Turf-to-Grass surfaces showed a similarly elevated rate (35.5%, 22/62 players). However, formal interaction testing was limited by small cell sizes in certain strata, particularly for hybrid surface transitions, resulting in model instability.

### 3.6. Sensitivity Analysis

To assess the robustness of our findings, we conducted a sensitivity analysis comparing only players with LE-NCIs (*n* = 175) to those who remained uninjured throughout the season (*n* = 267), excluding players with other types of injuries, and yielded similar patterns to our primary analysis. The adjusted odds ratios for surface transitions showed slightly stronger associations in the sensitivity analysis but remained statistically non-significant, suggesting that our findings were not substantially influenced by our approach to handling competing injury events (Supplementary Table S1).

## 4. Discussion

This study aimed to determine whether the transition in a home stadium turf type between a player’s final collegiate season and their rookie NFL season influenced the risk of lower extremity non-contact injuries (LE-NCIs) among first and second-round draft picks from 2012 to 2024. We hypothesized that transitions involving a change in surface type (e.g., Grass-to-Artificial and Artificial-to-Grass transitions) or involving hybrid surfaces would increase LE-NCI risk compared to a stable Grass-to-Grass transition. We also hypothesized that access to varied training surfaces in college would be protective and that the position group would moderate the transition effect. Our findings revealed that home stadium turf transitions from college to professional football were not significantly associated with LE-NCI risk during the rookie season. However, player position emerged as a critical risk determinant, with skill position players demonstrating 88% higher odds of injury compared to hybrid players (AOR = 1.88; 95% CI: 1.20–2.97; *p* = 0.006). These findings challenge our initial hypothesis of surface transition-mediated injury risk while highlighting the importance of position-specific biomechanical demands in rookie injury susceptibility.

Our null findings regarding surface transitions contrast notably with recent NFL injury surveillance data demonstrating higher overall injury rates on artificial turf surfaces. Venishetty et al. (2024) reported 60% higher odds of season-ending surgery for lower extremity injuries on artificial turf (OR = 1.60; 95% CI: 1.28–1.99) [1], while Jr et al. (2024) found that artificial surfaces were associated with 61.1% of knee ligament tears despite representing only 56.8% of games [2]. Similarly, Mack et al. (2019) documented a 16% higher rate of lower extremity injuries per play on synthetic turf compared to natural grass, with the effect being stronger for non-contact injuries [8].

However, these studies examined general surface effects across all players and career stages in the NFL, whereas our investigation specifically addressed transition effects during the rookie adaptation period. This distinction is crucial, as the mechanisms underlying general surface safety may differ from those affecting adaptation to surface changes. Our transition-focused approach represents a novel perspective that considers the prolonged neuromuscular adaptation challenges specific to career transitions rather than steady-state surface exposure effects.

The position-specific injury patterns we observed align closely with the established literature on NFL injury epidemiology. Previous research has consistently identified skill position players as having an elevated injury risk due to their high-speed cutting, acceleration, and deceleration demands [13]. Our finding of 88% higher odds for skill players compared to hybrid players corroborates this pattern and suggests that position-related biomechanical demands may outweigh surface transition effects in determining rookie injury risk.

The absence of significant transition effects despite documented biomechanical differences between surface types requires careful consideration. Kent et al. (2021) demonstrated that natural grass surfaces exhibit variable compliance (10–15 mm surface deformation) and rotational resistance (35–45 Nm rotational torque) [5], while artificial turf systems display 15–22% higher rotational traction and reduced compliance variability [6]. Thomson et al. (2015) showed that excessive rotational traction can “fix” the foot during directional changes, transferring injurious torque to ankle and knee joints [7].

Several factors may explain why these documented mechanical differences did not translate to increased transition-specific injury risk in our cohort. First, the rookie transition period involves numerous competing stressors—increased training loads, psychological pressures, and tactical complexity—that may overwhelm subtle surface-related effects [11,12]. Second, individual adaptation capacity likely varies substantially, with some athletes demonstrating greater neuroplasticity for surface adjustment than others.

Body mass may modify surface transition effects, though our findings suggest that movement patterns outweigh mass considerations. While heavier linemen (~300 pounds) theoretically experience greater ground reaction forces that could amplify surface stiffness effects, including higher rotational inertia during cutting maneuvers on high-traction surfaces [8], our results show that skill players (~190–210 pounds) had the highest injury risk. This suggests that movement velocity, frequency, and cutting demands characteristic of skill positions create high-magnitude rotational and shear forces regardless of surface type, potentially explaining why their injury risk remained elevated across all transition categories and why position-specific movement patterns emerged as the primary determinant rather than surface transitions.

Given our null findings for transition effects, it becomes important to understand why NFL organizations continue to invest in different surface types despite ongoing safety debates. Stadium surface selection involves complex decision-making that extends beyond injury prevention alone.

Climate and environmental constraints represent primary drivers of surface choice. Northern climate stadiums often necessitate artificial surfaces due to natural grass dormancy periods and growing season limitations. Teams like Green Bay, Chicago, and New England face seasonal temperature extremes that make year-round natural grass maintenance challenging. Additionally, indoor stadiums inherently require artificial systems due to limited natural light availability.

Economic and operational factors significantly influence surface decisions. Artificial turf installation could cost more than annual natural grass maintenance. However, artificial surfaces accommodate multi-use scheduling—including concerts, college games, and other events—generating additional revenue streams that natural grass cannot support due to damage susceptibility. This economic calculus often outweighs injury prevention considerations in organizational decision-making.

Performance consistency considerations also drive surface selection. Artificial turf provides uniform playing conditions regardless of weather, eliminating the field condition variability that can affect game outcomes. Some organizations prioritize this competitive consistency over potential injury risk differences, particularly when injury data remains debated within the scientific community.

Our findings have several important implications for NFL medical and performance staff. The significant position-specific injury risk highlights the need for targeted prevention strategies for skill position players. These athletes would benefit from enhanced neuromuscular training protocols focusing on cutting mechanics, landing strategies, and proprioceptive conditioning. Additionally, the nearly two-fold increase in injury odds for this position group suggests that they should receive priority attention in injury surveillance and load management protocols.

**Figure 3:**
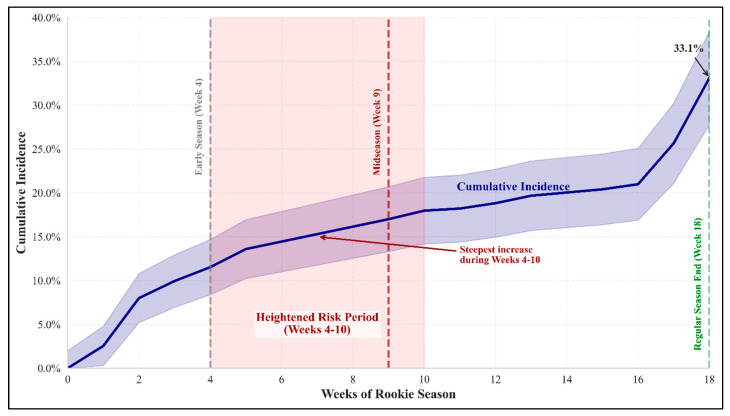
The cumulative incidence of lower extremity non-contact injuries during NFL rookie season. The figure illustrates the cumulative incidence of lower extremity non-contact injuries (solid blue line) with 95% confidence intervals (light blue shaded area) across the 17-week rookie season. Key timepoints are marked with vertical dashed lines: end of preseason (week 4, gray), midseason (week 9, red), and regular season conclusion (week 18, green). The red shaded area highlights the heightened risk period between weeks 4 and 10, during which the steepest increase in injury incidence occurs. By the end of the rookie season, the cumulative incidence reaches 33.1%, with a notable acceleration in injury rates during the final weeks (16–18) of the season. This temporal pattern suggests critical windows for targeted injury prevention strategies during the rookie transition period.

A cumulative incidence analysis revealed a substantial injury burden. The temporal clustering of injuries between weeks 4 and 10 provides a clear target for enhanced monitoring and intervention (Figure 3). This period corresponds to the transition from preseason to the regular season and the establishment of competitive rhythm. Medical staff should implement heightened vigilance during this window, potentially including increased soft tissue screening, modified training loads, and enhanced recovery protocols.

While surface transitions did not significantly affect the overall injury risk, the substantial injury burden (21.2% incidence) reinforces the importance of comprehensive rookie support programs. The cumulative incidence reaching 33.1% by season end demonstrates that rookie injury prevention must address multiple risk factors simultaneously rather than focusing solely on surface-related concerns.

### Limitations

Several important limitations must be acknowledged in interpreting our findings. Footwear–surface interactions represent a critical unmeasured variable that could modify surface transition effects. Different cleat designs (molded, detachable, and hybrid patterns) and materials interact differentially with surface types, potentially affecting traction and injury risk [7]. Individual footwear preferences and team equipment policies could substantially modify surface effects, yet these data were not available for our analysis.

Climate and temperature effects constitute another important limitation. Artificial turf surface temperatures can exceed 60 °C in direct sunlight compared to 25–30 °C for natural grass, affecting surface compliance and energy restitution [15].

Our retrospective design inherently limits the quality and completeness of injury classification, with 32% of injured players lacking definitive injury type documentation. Additionally, focusing on high-round draft picks limits generalizability to later-round selections and undrafted players, who may face different pressures and have different baseline characteristics.

The relatively small sample sizes for hybrid surface transitions (G-H: *n* = 28; A-H: *n* = 31) limited statistical power to detect meaningful differences for these increasingly common surface types. Similarly, our inability to test position-by-transition interactions due to sparse data in certain strata prevented a deeper exploration of mechanistic pathways.

Future investigations should incorporate prospective designs with detailed biomechanical assessments, environmental monitoring, and comprehensive footwear documentation. Larger multi-season cohorts would provide sufficient power to analyze hybrid surface effects and position-specific interactions. Additionally, studies should examine dose–response relationships between transition timing and injury risk, as well as investigate whether multiple surface exposures during college careers influence professional adaptation capacity.

Research incorporating individual movement analysis, surface temperature monitoring, and standardized footwear protocols would provide deeper mechanistic insights into the complex interplay between athletes, equipment, and environmental factors in determining injury risk during career transitions.

## 5. Conclusions

In conclusion, while the transition between collegiate and NFL home stadium surfaces was not identified as an independent risk factor for overall LE-NCI occurrence among high-round rookie draft picks in this cohort, player position and the timing within the rookie season are critical factors. Skill position players face a significantly higher risk, and a period of heightened vulnerability exists from the early- to mid-season. These findings emphasize the need for position-specific and temporally targeted injury prevention strategies to support athlete health and performance during the demanding transition into professional football.

## Figures and Tables

**Table 1:** Baseline characteristics by lower extremity non-contact injury status.

Characteristic	Category	No LE-NCI (*N* = 651)	Yes LE-NCI (*N* = 175)	*p*-Value
Surface Transition	Grass–Grass	151 (23.2%)	47 (26.9%)	0.175
	Grass–Hybrid	26 (4.0%)	2 (1.1%)	
	Grass–Turf	157 (24.1%)	44 (25.1%)	
	Turf–Grass	147 (22.6%)	47 (26.9%)	
	Turf–Hybrid	27 (4.1%)	4 (2.3%)	
	Turf–Turf	143 (22.0%)	31 (17.7%)	
College Training Access	No	191 (29.3%)	52 (29.7%)	0.997
	Yes	460 (70.7%)	123 (70.3%)	
Position Group	Hybrid	143 (22.0%)	34 (19.4%)	<0.001
	Linemen	259 (39.8%)	46 (26.3%)	
	Quarterback	46 (7.1%)	7 (4.0%)	
	Skill	203 (31.2%)	88 (50.3%)	

Baseline characteristics of study cohort stratified by lower extremity non-contact injury (LE-NCI) status. Data are presented as *n* (%). *p*-values were derived from Chi-square or Fisher’s exact tests comparing distributions between No LE-NCI and Yes LE-NCI groups. Abbreviations: G-G, Grass-to-Grass; G-H, Grass-to-Hybrid; G-T, Grass-to-Artificial Turf; T-G, Artificial Turf-to-Grass; T-H, Artificial Turf-to-Hybrid; T-T, Artificial Turf-to-Artificial Turf. College training access category of “Yes” indicates access to multiple surface types during final college season, while “No” indicates access to only one type.

**Table 2:** Lower extremity non-contact injury risk by position group and surface transition.

Position	Adjusted Odds Ratio (95% CI) vs. Hybrid	Transition	LE-NCI/Total	Injury% (95% CI)
Hybrid	1.00 (reference)	All Transitions	34/177	19.2 (14.0–25.4)
		Grass-to-Grass	12/44	27.3 (16.3–41.2)
		Grass-to-Turf	10/43	23.3 (12.8–37.3)
		Turf-to-Grass	6/44	13.6 (6.0–26.5)
		Turf-to-Turf	6/34	17.6 (8.1–32.6)
		Grass-to-Hybrid	0/4	0.0 (0.0–54.7)
		Turf-to-Hybrid	0/8	0.0 (0.0–35.4)
Linemen	0.76 (0.47–1.25)	All Transitions	46/305	15.1 (11.5–19.4)
		Grass-to-Grass	6/72	8.3 (3.7–16.1)
		Grass-to-Turf	10/68	14.7 (7.9–24.6)
		Turf-to-Grass	17/71	23.9 (15.4–34.6)
		Turf-to-Turf	12/74	16.2 (9.3–25.9)
		Grass-to-Hybrid	1/12	8.3 (0.4–36.0)
		Turf-to-Hybrid	0/8	0.0 (0.0–35.4)
Quarterback	0.64 (0.26–1.54)	All Transitions	7/53	13.2 (6.2–24.0)
		Grass-to-Grass	2/12	16.7 (2.9–45.9)
		Grass-to-Turf	3/10	30.0 (8.7–61.3)
		Turf-to-Grass	2/17	11.8 (2.0–34.4)
		Turf-to-Turf	0/10	0.0 (0.0–29.2)
		Grass-to-Hybrid	0/2	0.0 (0.0–65.9)
		Turf-to-Hybrid	0/2	0.0 (0.0–65.9)
Skill	1.88 (1.20–2.97)	All Transitions	88/291	30.2 (25.2–35.7)
		Grass-to-Grass	27/70	38.6 (27.6–50.5)
		Grass-to-Turf	21/80	26.2 (17.6–36.8)
		Turf-to-Grass	22/62	35.5 (24.2–48.1)
		Turf-to-Turf	13/56	23.2 (13.6–35.9)
		Grass-to-Hybrid	1/10	10.0 (0.5–40.3)
		Turf-to-Hybrid	4/13	30.8 (10.7–59.0)

Number of LE-NCI cases, total players, injury percentage, and odds ratios for each position group and surface transition, with odds ratios calculated relative to hybrid position group, are displayed. Note: LE-NCI = Lower Extremity Non-Contact Injury; G = Grass; T = Turf; H = Hybrid. Odds ratio calculated compared to hybrid position group (reference).

## Data Availability

The raw data supporting the conclusions of this article will be made available by the authors upon request.

## Supplementary Materials

The following supporting information can be downloaded at: https://www.mdpi.com/article/10.3390/healthcare13121415/s1, Table S1: Sensitivity analysis: Adjusted odds ratios from logistic regression comparing lower extremity non-contact injury (LE-NCI) cases to uninjured players only. Results compare only players with LE-NCI (n=175) to those who remained uninjured (*n* = 267), excluding players with other types of injuries. Model includes surface transition, college training access, position group, and draft cohort. Significant (*p* < 0.05), bolded, and borderline (*p* < 0.10) ‘†’ associations are indicated. Note: AOR = Adjusted Odds Ratio; CI = Confidence Interval.

## Appendix A

**Table A1:** Multivariable logistic regression models for lower extremity non-contact injury risk.

Variable	Model 1 AOR (95% CI)	Model 1 *p*-Value	Model 2 AOR (95% CI)	Model 2 *p*-Value
Surface Transition (ref: Grass-to-Grass)				
Grass-to-Hybrid	0.254 (0.06–1.12)	0.071	0.254 (0.06–1.13)	0.071
Grass-to-Turf	0.877 (0.54–1.41)	0.589	0.877 (0.54–1.41)	0.591
Turf-to-Grass	1.087 (0.68–1.75)	0.731	1.075 (0.66–1.76)	0.775
Turf-to-Hybrid	0.437 (0.14–1.33)	0.145	0.433 (0.14–1.33)	0.143
Turf-to-Turf	0.741 (0.44–1.25)	0.260	0.734 (0.43–1.25)	0.257
Position Group (ref: Hybrid)				
Linemen	0.765 (0.47–1.25)	0.284	0.764 (0.47–1.25)	0.284
Quarterback	0.635 (0.26–1.54)	0.314	0.636 (0.26–1.54)	0.315
Skill	**1.885 (1.20–2.97)**	**0.006**	**1.884 (1.20–2.97)**	**0.006**
Draft Cohort (ref: 2012–2015)				
2016–2019 (Hybrid Transition)	1.207 (0.78–1.86)	0.393	1.209 (0.78–1.87)	0.390
2020–2024 (Turf 4.0/Grass)	0.969 (0.63–1.49)	0.885	0.974 (0.63–1.50)	0.904
College Training Access (ref: No)				
Yes	-	-	0.968 (0.65–1.45)	0.874

Multivariable logistic regression models evaluating the association between surface transition, position group, draft cohort, and college training access with lower extremity non-contact injury (LE-NCI) risk. Model 1 includes surface transition, position group, and draft cohort; Model 2 additionally includes college training access. Significant associations (*p* < 0.05) are bolded. Note: AOR = Adjusted Odds Ratio; CI = Confidence Interval.
