# Supplementary material for: College-to-NFL Stadium Turf Transitions as a Risk Factor for Lower Extremity Non-Contact Injuries in Rookie Players: A 13-Year Cohort Analysis
Source: Healthcare (Basel). 2025 Jun 13;13(12):1415. doi: 10.3390/healthcare13121415 (PMC12193070; doi:10.3390/healthcare13121415)
Supplement: Supplementary file 1 [file healthcare-13-01415-s001.zip › healthcare-3649690-supplementary.pdf]

SUPPLEMENTARY

Variable	AOR (95% CI)	P-value
**Surface Transition (ref: Grass-to-Grass)**		
**Grass-to-Hybrid**	0.209 (0.044–1.002)†	0.050†
**Grass-to-Turf**	0.945 (0.536–1.665)	0.844
**Turf-to-Grass**	1.141 (0.635–2.050)	0.659
**Turf-to-Hybrid**	0.308 (0.090–1.057)†	0.061†
**Turf-to-Turf**	0.822 (0.440–1.533)	0.537
**College Training Access (ref: No)**		
**Yes**	1.111 (0.696–1.772)	0.660
**Position Group (ref: Hybrid)**		
**Linemen**	0.740 (0.425–1.288)	0.287
**Quarterback**	0.637 (0.241–1.685)	0.363
**Skill**	**2.105 (1.235–3.590)**	**0.006**
**Draft Cohort (ref: 2012–2015)**		
**2016–2019 (Hybrid Transition)**	0.601 (0.357–1.015)†	0.057†
**2020–2024 (Turf 4.0/Grass)**	0.651 (0.384–1.103)	0.111

Supplementary Table S1. Sensitivity analysis: Adjusted odds ratios from logistic regression comparing lower extremity non-contact injury (LE-NCI) cases to uninjured players only. Results compare only players with LE-NCI (n=175) to those who remained uninjured (n=267), excluding players with other types of injuries. Model includes surface transition, college training access, position group, and draft cohort. Significant ($p < 0.05$), bolded, and borderline ($p < 0.10$) '† ' associations are indicated. **Note:** AOR = Adjusted Odds Ratio; CI = Confidence Interval.
